# Duration of Care in Early Intervention in Psychosis Services: A Multi‐Perspective Qualitative Study

**DOI:** 10.1111/eip.70199

**Published:** 2026-07-09

**Authors:** Michelle Rickett, Tom Kingstone, David Shiers, Paul French, Belinda Lennox, Mike Crawford, Ryan Williams, Jonathan Woodward, Carolyn Chew‐Graham

**Affiliations:** ^1^ Keele University Staffordshire UK; ^2^ Pennine Care NHS Foundation Trust Greater Manchester UK; ^3^ University of Oxford Oxford UK; ^4^ Imperial College London UK; ^5^ University of Sheffield Sheffield UK

**Keywords:** delivery of healthcare, discharge planning, patient care, psychotic disorders, qualitative methods, severe mental illness

## Abstract

**Introduction:**

Early Intervention in Psychosis (EIP) services in England are commissioned to provide up to 3 years' support to people experiencing first episode psychosis, but the optimal duration remains debated. This qualitative study aimed to explore how decisions about EIP duration are made and experienced by multiple stakeholders.

**Methods:**

Qualitative longitudinal study comprising semi‐structured interviews with EIP Service Users (SUs), carers, EIP practitioners, GPs, and mental health service commissioners; follow‐up interviews with SUs 6–11 months later. Data were collected between September 2022 and September 2024 and informed by information power. Data were thematically analysed by a multidisciplinary team. Patient and carer involvement and engagement were integral to the study.

**Results:**

The 3‐year time limit of contact with EIP teams was thought to work well when SUs felt supported and ready for discharge, but constrained shared decision making when they did not. Some participants described early discharge before 3 years, either as a negotiated decision based on wellness and readiness to move on or as a consequence of poor engagement. Others reported extended care beyond 3 years in response to individual needs or delays in transfer to Community Mental Health Teams (CMHTs). This flexibility in duration varied across teams and trusts. Well‐managed, relational care was viewed as key to effective discharge.

**Conclusion:**

Flexible, person‐centred episodes of care and collaborative discharge planning, supported by effective coordination between EIP, primary care, and CMHTs, are essential to sustaining relational practice and ensuring smoother transitions.

## 1 Introduction

Early Intervention in Psychosis (EIP) services offer multidisciplinary treatment in the community to people experiencing a first episode of psychosis. The lifetime prevalence of psychosis is estimated at 4 per 1000 of the population in England (Kirkbride et al. 2013). Psychosis can have wide‐ranging and profound implications for individuals' quality of life, including effects on physical health, cognition, social functioning, social inclusion, education and employment (Mason et al. 1995; Meltzer 2002). Schizophrenia and other psychotic disorders are major contributors to the global burden of disease, accounting for around £2 billion per annum in NHS expenditure (Mangalore and Knapp 2007; Murray and Lopez 1996).

In the United Kingdom (UK), EIP services offer a three‐year package of treatment. The rationale for this time‐limited model is that early intensive treatment could preclude the need for such intensive treatment on an ongoing basis (i.e., a secondary prevention approach) (Bertolote and McGorry 2005; Morgan et al. 2014). However, National Institute for Health and Care Excellence (NICE) 2020 guidance (CG178) recognise that 2–3‐years of interventions is not optimal for everyone and some may benefit from extended time under EIP to consolidate improvement that has been achieved (National Institute for Health and Care Excellence (NICE) 2020).

People can be referred into EIP services through General Practitioners (GPs) in primary care (the most common route), Community Mental Health Teams, acute or emergency settings, or self‐referral (NHS England 2023). At the end of their time under EIP, service users (SUs) are discharged back to primary care if they are stable or transferred to Community Mental Health Teams if they need ongoing specialist mental health support (Puntis et al. 2024). Decisions regarding continuation or discharge from EIP are made within multidisciplinary EIP teams.

This qualitative study aimed to understand and contextualise the duration of EIP care provided—including individual variation, differences in processes of care, discharge planning and shared decision‐making—from the perspectives of service users (SUs), carers, healthcare practitioners and commissioners. This was part of the National Institute for Health and Care Research (NIHR) programme ‘EXTEND: personalised care for early psychosis’, which explored the impact of EIP duration on service user outcomes (EXTEND Website 2025).

## 2 Methods

We conducted in‐depth, semi‐structured interviews with EIP SUs at point of discharge or shortly after discharge (excluding those lacking capacity of expressing suicidal ideation); carers of SUs who were at point of discharge or shortly after; EIP healthcare practitioners (HCPs), General Practitioners (GPs), and commissioners of mental health services. SUs and carers were recruited using both purposive and convenience sampling. Potential participants were identified by EIP teams within Mental Health Trusts (MHTs) across England, or self‐identified by responding to a study flyer shared via social media (XR), mental health networks, support groups and charities. EIP practitioners and managers were identified through participating MHTs; and GPs and commissioners were identified through professional networks and snowballing (Noy 2008). An information sheet and ‘consent to contact’ form were used. Interested individuals were contacted by the study researcher who checked their eligibility against inclusion criteria and invited them to participate in an interview. Consent forms were completed before or at the beginning of the interview.

Topic guides were developed by the qualitative research team, alongside lived experience co‐investigators and our patient and carer advisory group, ‘EXTENDing’. Topic guides for all four groups of interviews explored experiences and views of EIP services; duration of care; and discharge planning and decision‐making. The topic guide for SU follow‐up interviews focused on care after discharge; support needs and gaps; and additional reflections on EIP. The guides were modified iteratively alongside data generation and analysis. Data collection was informed by information power; an alternative concept to saturation in qualitative research that involves pragmatic judgements based on aims, specificity, theory, dialogue and analysis (Malterud et al. 2016).

We took a reflexive thematic approach to analysis, which involved reading and re‐reading transcripts, generating and organising codes, and constructing themes (Braun et al. 2023). Transcripts were grouped and analysed by participant type initially, then cross‐cutting themes were generated. The lead researcher (MR) coded the data, led analysis, and met regularly with team members representing a range of disciplinary backgrounds to refine interpretation and agree on the key themes.

### 2.1 Patient and Public Involvement and Engagement (PPIE)

Patient and carer involvement and engagement were integral to our research process. We met regularly with our two EXTEND PPI co‐investigators and the EXTENDing group who co‐designed topic guides and public‐facing documents, advised on recruitment strategies and helped to identify, clarify and deepen findings during analysis‐focused meetings with the research team and via email feedback.

## 3 Results

We conducted semi‐structured interviews with 16 SUs at, or soon after discharge; 14 carers; 24 EIP practitioners; 8 GPs; and 6 mental health commissioners. Twelve SU follow‐up interviews were conducted: 11 of these were conducted 6–8 months after the first interview and one SU was re‐interviewed a second time at 11 months post‐discharge, after delayed discharge to a CMHT. Interviews lasted between 20 and 50 min and were conducted online or by telephone (Tables 1, 2, 3, 4).

**TABLE 1:** SU demographics.

Participant ID	Gender	Age range (years)	Ethnic background (self‐described)	Living circumstance	Second interview
SU1	Female	31–40	White British	Lives with partner and children	✓ (reinterviewed twice as SU was still waiting to be discharged at first follow up)
SU2	Female	21–30	White British	Lives alone	✓
SU3	Female	21–30	White and Black Caribbean	Lives with partner and child	✓
SU4	Female	21–30	Mixed/multiple ethnicity	Lives with father	✓
SU5	Female	41–50	White Polish	Lives with partner and child	✓
SU6	Female	21–30	White British	Lives with father	✓
SU7^a^	Female	61–70	White British	Lives with partner	✓
SU8	Female	51–60	Black Caribbean	Lives alone	✓
SU9	Male	41–50	Black British	Lives with partner	☒
SU10	Female	61–70	White British	Lives with partner	☒
SU11^a^	Male	21–30	White British	Lives alone	✓
SU12	Male	41–50	White British	Lives with partner and children	☒
SU13	Male	41–50	White British	Lives alone	✓
SU14	Male	31–40	White British	Lives with parents	✓
SU15	Male	31–40	White British	Lives alone	☒
SU16	Male	41–50	White Australian	Lives with partner	☒

^a^ Dyadic interviews with carer.

**TABLE 2:** Carer demographics.

Participant ID	Gender	Relationship to person with Psychosis	Age range (years)	Ethnic background (self‐described)
CAR1	Male	Brother	21–30	Asian
CAR2	Male	Friend	21–30	Black British
CAR3	Female	Niece	21–30	Indian British
CAR4	Female	Mother	61–70	White British
CAR5^a^	Male	Husband	51–60	White British
CAR6	Male	Grandchild	21–30	Black British
CAR7	Female	Mother	51–60	White British
CAR8	Male	Partner	61–70	White British
CAR9	Female	Former partner	41–50	Bangladeshi
CAR10	Female	Mother	41–50	White British
CAR11	Female	Mother	41–50	White British
CAR12	Female	Mother	51–60	White British
CAR13^a^	Female	Mother	61–70	White British
CAR14	Female	Daughter‐in‐law	31–40	White Other (Italian)

^a^ Dyadic interviews with SU.

**TABLE 3:** GP background.

Participant ID	Gender	Years as GP	Geography (self‐described)
GP1	Female	10	Inner city
GP2	Male	5	Urban
GP3	Male	5 years as partner	Rural
GP4	Female	25	Participant worked across several practice in different locations
GP5	Male	11 years as partner	Suburban
GP6	Female	19	Urban
GP7	Female	13	Semi‐rural
GP8	Female	4	Urban

**TABLE 4:** EIP practitioners/manager/commissioner background.

Participant ID	Gender	Job title
HCP1	Female	Clinical Lead, Early Intervention in Psychosis
HCP2	Female	EIP Team Manager
HCP3	Female	EIP Team Manager
HCP4	Female	Care co‐ordinator/Mental Health Nurse
HCP5	Male	Consultant Psychiatrist
HCP6	Male	Advanced Clinical Practitioner/Social Worker
HCP7	Female	Care co‐ordinator/Mental Health Nurse
HCP8	Female	Community psychiatric nurse
HCP9	Male	Consultant Psychiatrist
HCP10	Male	Consultant Psychiatrist
HCP11	Female	EIP Team Manager
HCP12	Male	Psychologist
HCP13	Male	Case manager
HCP14	Male	EIP Team Manager
HCP15	Female	Clinical Nurse specialist
HCP16	Female	EIP Team Manager
HCP17	Male	Principal Psychologist
HCP18	Female	Principal Psychologist
HCP19	Male	Community Psychiatric Nurse
HCP20	Female	Clinical Lead, Early Intervention in Psychosis
HCP21	Female	Care co‐ordinator/Mental Health Nurse
HCP22	Male	Community Psychiatric Nurse
HCP23	Female	Peer support worker
COM1	Male	Senior Programme Manager
COM2	Male	Senior Programme Delivery Lead
COM3	Female	Associate Director for Specialist Services
COM4	Female	Senior Transformation Manager, Mental Health
COM5	Female	Interim Head, Mental Health, Learning Disabilities and Autism
COM6	Female	Trust EIP Lead

Our findings are reported under four key themes: time‐limited care; flexible approaches—early discharge; flexible approaches—extended care; managed transition. Illustrative quotes are presented with participant identifiers (e.g., SU3).

### 3.1 Time‐Limited Care

Most SUs reported having received the standard 3 years' EIP care. SUs felt that this worked well when discharge was planned collaboratively, care coordinators were supportive, and they felt ready, prepared, and equipped with the tools they needed to manage independently.

I think having a discussion with my care coordinator, and a chat yesterday when I had my consultant appointment, it was just a joint view, really…Yeah, it's all been coordinated and I've had my decision in that. (SU1)

I think the length is just right. I don't think there is anything more that I feel that they can offer me. They've given me all the tools to sort of help myself as well. (SU8)

However, the time‐limited approach was seen as a barrier to shared decision‐making for those who didn't feel ready for discharge, leading some to describe feelings of disempowerment and abandonment.

They just, you know, they couldn't keep me longer so, that was reason, yeah. So, they asked me if I agree with that, I said, ‘yes,’ I didn't have a choice I think, you know. (SU5)

She's just a bit of flotsam in the system as far she's concerned, I think. I mean they did try and involve her in all decision‐making but when the decision is—‘You're leaving us and you're going to the CMHT,’ and there's no decision to be made and there's no choice—you can flannel and you can dress it up, but it is what it is. (CAR4)

Healthcare Practitioners (HCPs), SUs and carers felt that strict adherence to the three‐year limit could risk relapse for some people due to the loss of the relationship with the care coordinator and less intensive care, as well as loss of progress made during EIP:

The people that are coming up to that three years they're relapsing and I don't know whether because the safety net's been taken away. (HCP4)

It's like a weird feeling it's been so long just having someone there as like a bit of a comfort blanket and then all of a sudden they're not going to be there anymore and you just think oh no… And then (care coordinator) used to think that it may have been something to do with me having another breakdown (after discharge), knowing that I was losing that support, as well as a number of other things. (SU2)

Related to this, most EIP managers and commissioners identified the tension between resource/caseload pressures and responding to individual need.

I think that there's been times when people have been moved on when we could probably justify keeping them for longer, but again it's that tension between if it sets a precedence… then if that becomes the norm that then has an impact on caseload size. (HCP1)

### 3.2 Flexible Approaches: Early Discharge

Participants reported that some SUs were discharged from EIP before 3 years, usually through negotiated agreement between SUs and EIP practitioners, reflecting wellness and readiness, access to and completion of therapies, or personal preference. In these cases, early discharge was experienced positively:

He's doing much, much better now, his usual life has been pretty much restored, he's feeling quite satisfied with how things are. (CAR1)

It feels like there's a sense of like we got as far as we can get to at this stage with people. Maybe they recovered, maybe they've stopped taking meds, maybe they're continuing and happy to be taking medication They might've had therapy, might not have felt they needed that. Maybe they're back at work. …So I guess in a lot of cases I like to think that's probably quite negotiated. (HCP12)

Conversely, some early discharges resulted from lack of engagement and challenges in relationship‐building.

They stop engaging or they don't really engage, got quite an avoidant, you know, style. I guess often times it's over‐represented in psychosis isn't it? I think people are more withdrawn at times so yeah is there that engagement issue. (HCP17)

One GP voiced frustration about the decision to discharge SUs due to non‐engagement, suggesting that ‘not engaging is probably a sign that they're unwell, not a sign that all is hunky‐dory.*’ (GP6)*.

### 3.3 Flexible Approaches: Extended Care

#### 3.3.1 Capacity and Waiting Lists

One of the most common reasons for extending EIP care beyond 3 years, cited by EIP HCPs, was lack of capacity in receiving CMHTs. SUs who were not felt to be suitable for discharge to primary care remained under EIP care awaiting transfer:

So obviously if someone has ongoing support needs they can't be discharged back to primary care and (if) we can't transfer them to a relevant team, then we can't discharge them. So, they will have to stay under us. (HCP13)

The uncertainty surrounding these delays could feel worrying and destabilising for SUs and carers:

It doesn't feel very secure and safe. Everything is just up in the air at the minute. I've felt quite anxious about it. (SU3 follow up interview)

#### 3.3.2 Personalised Approaches to Extending Care

Most EIP HCPs felt that some SUs would benefit from extended care under EIP, but this was often not possible due to the time‐limited approach.

There was definitely a group of people who at that three‐year point looked like they had the potential for more recovery, if they had the same level of input that we're providing them. But we're only funded to three years. (HCP13)

Care was occasionally reported to be extended in practice, often through the advocacy and experience of care coordinators, who could play an important role in decision‐making:

I've got a bit of a reputation, I do. So, I mean, obviously I've been around a long time in the team, so I've probably got more examples to draw on (of extending care) than others have. I've had a couple where I've fought for them to be extended, for various reasons…Just things that come up that you think, ‘Oh, I couldn't possibly discharge her now, this is not a good time,’ …. (HCP20)

EIP care was sometimes extended to allow completion of EIP therapies, which could be delayed by waiting lists or SUs only being ready to engage with therapies towards the end of the 3 years:

I think if there's a justification to extend somebody because they might not have been able to access part of the full EI care package for whatever reason, and that person was ready and willing to be engaged in that, and then we think that that would be of benefit to them then they'll extend their care. (HCP1)

SUs who were particularly unwell or unstable were also reported to stay under EIP beyond 3 years to avoid exacerbating their symptoms:

Well, there were several times they felt he was still too ill to move on. And then they really were going to move him and then COVID and that really got in the way. So, they kept him all that time and then he ended up ill again in hospital. So, it's just been extended and extended. (CAR7)

Significant life events and social factors (e.g., pregnancy, bereavement, changes in work/education, housing crises) could lead to delayed discharge from EIP service for some people:

As I was due to be discharged and transferred to the community mental health team, I was pregnant and…my due date kind of postponed it and they wanted to keep an eye on me because of the risk you know, postpartum. So, yes, that's the reason why like I have been allowed that extension. (SU3)

So, we've had somebody recently who was due to be discharged a couple of months ago, but they had a housing crisis at that time. …so we agreed to kind of continue to support for a period of time… (HCP3)

In a small number of cases, care was extended for reasons related to neurodiversity—e.g., to facilitate formal assessment or to ease the transition to other services.

She was not too far away from coming up to the three‐year period when we kind of put everything together and wondered whether she might be on the autistic spectrum. So, she was referred for an assessment, she agreed to that, and the service here… agreed to fast track her assessment while she was involved with services. So that delayed her service by about four or five months, while we kind of got through that process and supported her with that, and she was given a diagnosis of autism. (HCP20)

This personalised approach was appreciated by SUs with neurodiverse conditions:

They've been making sure that I'm not getting too overwhelmed about it. I have been able to ask questions. I'm not really good with change, so they've been taking it slow with us, which I'm really grateful for. (SU6)

However, in other cases, SUs with suspected neurodiverse conditions were discharged at 3 years and then struggled to receive timely assessment and support.

I had two care coordinators. The first one said, ‘I did suspect you probably (have autism), but I'm not too sure’ and that wasn't really taken that far and then she left basically. And then the new care coordinator took over and then straight away just said, ‘Yeah, I'm making you a referral’ and I've literally been waiting about three and a half years for it. So, it was only the other week that I've actually had (the diagnosis). So that's a good year and a bit out of the care of the early intervention team. (SU13)

In a minority of cases, duration of EIP care was reported to be extended due to collaborative decision making by care coordinators and SUs. This helped SUs feel supported and listened to.

I think the reason (for the extension) was there was lots of change going on and also they felt that I needed it…More importantly, I felt that I needed it as well. I felt that they did listen to me and responded very appropriately and very positively " (SU16)

Some HCPs expressed a desire for more transparent procedures to formalise such decisions:

I mean, length of care, I would love for there to be a kind of an agreed facility to do this for those people that you do feel need a bit longer…. if I had known we could've offered another year, then I could've said, ‘Let's extend it for a year, this is going to be your new date, that's what we're working towards in that piece of time’

#### 3.3.3 When Extending Care Isn't Enough…

Some carers of SUs with severe, enduring illness felt that even extending EIP care wasn't enough, and that their loved ones needed ongoing high levels of support and rehabilitation that weren't adequately provided when they were discharged to CMHTs:

If somebody's got a diagnosis of schizophrenia, which is lifelong, that's very different to somebody that's experiencing a one‐off psychosis. She needs long‐term support. (CAR11)

HCPs also felt that some SUs with severe symptoms and complexity needed more than an extension of care:

If you are from…socially oppressed, social economic, cultural background or you have a complex sort of neurodevelopmental conditions as well intermixed with that then you're often spending that three years just trying to get over the first hurdle to get them engaged in that process…You get this kind of like intense group of people that need much more support and I think they are a lot of the patients that are revolving door admission patients and needing assertive outreach and needing that kind of, that more push of engagements, really… it's probably kind of a combination of assertive outreach and extension that's needed. (HCP6)

### 3.4 Managed Transition

SUs, carers and EIP HCPs all felt that the close, supportive relationships formed within EIP were key to its success:

The relationship and the attachment they have to us as the service and clinicians is that kind of key bedrock of what helps people. *(HCP17)*

The EIP team…especially the care coordinator…they get to know you, your family setup and everything. I think that, for me, was really important as well. Just understanding that I'm more than what I've just been through…Just make you feel like you've got that hope that you are going to get better. (SU1)

SUs and carers expressed concern about the loss of these relationships, which would not be replicated after discharge. This was an issue irrespective of duration of care:

It's been so long just having someone there as like a bit of a comfort blanket and then all of a sudden they're not going to be there anymore…so obviously not having that there was pretty worrying. (SU2)

One carer felt that a *‘gentler transition’ (CAR8)* would have led to a more positive experience of discharge for him and his loved one.

## 4 Discussion

Our research found substantial variation across EIP teams in how flexibly the three‐year period of care is applied. Decisions to extend care are influenced not only by clinical need but also capacity and caseload pressures within EIP and CMHTs, as well as care coordinator expertise and advocacy. This suggests that SUs and carers can experience inequitable access to extended care and inconsistent pathways. Person‐centred, relational care is key to the EIP model and this cannot be fully achieved if there is a lack of flexibility or shared decision making in relation to duration of care.

Many EIP HCPs feel that there are circumstances in which extended care can improve SU outcomes and experiences both within EIP and after discharge. SUs and carers reported positive experiences when extensions were negotiated and responsive to individual needs and circumstances. However, the absence of formal guidelines leaves such decisions dependent on local discretion and judgement. For some SUs with more severe illness, an extended period of EIP still does not meet their needs.

### 4.1 Strengths and Limitations

Our study incorporated perspectives from diverse participants and involved people with lived experience throughout the research process. The use of information power to aid data collection added rigour to our analysis, ensuring that we focused on generating the optimal volume, quality, and specificity of data to answer our research questions.

Only three of the 16 SUs interviewed were discharged to CMHTs and just over half were women, which may not fully reflect the EIP population. Further research exploring the experiences of male SUs and those discharged to CMHTs would be important. SUs with severe symptoms were less likely to participate in this research. However, several carers reported that they provided care for people with severe illness and gave insight into these experiences. Our research may slightly over‐represent the experiences of older SUs but there is increasing evidence that (since the removal of upper age limits for EIP services in the UK) service users aged > 35 make up a substantial proportion of EIP caseloads (Williams et al. 2026; Taylor et al. 2023). Some of our carer participants were caring for younger people and HCPs also discussed younger SUs under their care.

### 4.2 Comparison With Literature

We believe this is the first qualitative study to examine how EIP duration of care and discharge are negotiated and experienced in practice, from the perspectives of SUs, carers and healthcare practitioners. It reinforces previous qualitative studies on SU experiences of discharge from EIP, highlighting the centrality of relational care within EIP and the sense of loss SUs can feel when losing these relationships as well as the need for advance planning (Loughlin et al. 2019; Lester et al. 2012; Rickett et al. 2025), Previous research has also shown that continuity of care within EIP and strong relationships with care coordinators lead to improved outcomes (Williams et al. 2025; Lester et al. 2009; Adair et al. 2005).

The literature shows that there are a range of illness trajectories following a first episode of psychosis, and a significant proportion of people do not reach remission nor recovery criteria after their FEP (Lally et al. 2017). Our carer narratives in particular highlight the challenges of navigating care and discharge for SUs with ongoing severe symptoms. Evidence suggests that early access to psychiatric rehabilitation can be beneficial for this group, but we found little evidence of this being offered (Kuipers 2014).

Previous literature suggests that there is unmet need for people who have a first episode of psychosis and suspected neurodivergence (Nikolić et al. 2024). Our research shows that personalised approaches to duration of care and discharge are particularly impactful for this group of SUs.

## 5 Conclusion

This research highlights the benefits of personalising EIP duration of care to improve patient experiences and outcomes, and identifies circumstances where extended care has a positive impact. We suggest that collaborative decision‐making and planning around discharge from EIP and continuity of care post‐discharge are key to achieving optimal experiences for SUs and carers, irrespective of the duration of formal EIP involvement.

In terms of implications for practice, we suggest that relational care should be promoted through planned, supported discharge processes, including shared care protocols and clear communication between EIP, primary care and CMHTs. Practitioners should promote consistency in decision‐making about duration and discharge planning, including considering specific cases where transition needs may differ. EIP SUs and carers should be actively involved in discussions and decisions around the duration of their care. At a service and system level, mental health commissioners, policy makers, and EIP teams should embed SU and carer perspectives within the commissioning and delivery of EIP services, including in relation to duration of care and EIP discharge.

## Funding

This study/project (EXTEND: Personalised Care for Early Psychosis: https://www.psych.ox.ac.uk/research/extend) is funded by the National Institute for Health and Care Research (NIHR) under its Programme Grants for Applied Research (reference: NIHR203277). Chew‐Graham is part‐funded by the NIHR Applied Research Collaboration West Midlands. The views expressed are those of the author(s) and not necessarily those of the NIHR or the Department of Health and Social Care.

## Ethics Statement

Research ethics approval was obtained in September 2022 from the North of Scotland Research Ethics Committee (reference: 22/NS/0113) and the Health Research Authority.

(Integrated Research Application System ID: 313927).

## Supporting information


**Data S1:** Supporting Informations—Topic guides.

## Data Availability

Anonymised data is available on reasonable request.
