# Supplementary material for: Duration of Care in Early Intervention in Psychosis Services: A Multi‐Perspective Qualitative Study
Source: Early Interv Psychiatry. 2026 Jul 9;20(7):e70199. doi: 10.1111/eip.70199 (PMC13348009; doi:10.1111/eip.70199)
Supplement: Supplementary file 1 — Data S1: Supporting Informations—Topic guides. [file EIP-20-0-s001.docx]

**Supplementary materials – Topic guides**

**TOPIC GUIDE _ SERVICE USER (SU) FIRST INTERVIEW**

**Introductions**

Answer any questions from participant

Complete consent process – including consent to record.

Ask for details of GP surgery and explain that this is needed in case the participant becomes distressed and needs support contacting their GP (as in PIL)

**Background information**

Gender, age, ethnic background, family circumstances, housing, employment, other physical and mental health diagnoses

**Interview topics to be covered**

1. When were you in an EIP service?
2. How long were you/have you been in the EI service?
   1. Explore experiences which led to original referral. Can you tell me how you came to be referred to the EIP service? How was that experience?
   2. Explore current challenges – physical health, mental health, life circumstances
   3. Explore hopes for the immediate future
3. Can you outline the support/treatment you have received from the EI service?
   1. What help did they provide?
      1. Explore talking treatments, case manager support, medication, other
   2. Who was most helpful and why?
      1. Explore talking treatments, case manager support, medication
   3. Did you develop any physical health problems during your time under EI service and did EI service help you manage them?
   4. Did you have routine physical checks during your time under EI service? And were these within the EIP service or from your GP?
   5. Did you have any contact with your GP / practice whilst you were under care of EiP service?
   6. How long were you under the care of the EI service?
4. Did your close supporters/loved ones receive any support from EI service?
   1. If so, what? (at time of admission to service, when in service, to plan discharge)
   2. If not, what might have been useful?
5. How do you feel EI service has helped you?
   1. Mental health
   2. Physical health
   3. Has it helped you feel equipped to move forward with your life? If so, in what ways?
6. What preparation and support did you receive from the EI service for your discharge?
   1. How were you involved in the decision about discharge from EI service? How did you feel about being discharged?
   2. How were you involved in planning your discharge and the services you might have following discharge from the EI service? What factors were important to you?
   3. Have you been supported in managing your health around discharge? (explore mental and physical health)
   4. Were your supporters/loved ones involved in planning your discharge?
   5. Was your GP involved in discharge planning?
7. What services have been put in place to support you following discharge?
   1. Explore health support - CMHT (if involved) or other psychiatric support; GP; for both physical and mental health
   2. Explore role of third sector organisations, social prescribing (‘community-based activities’), housing support, education, work, finance, social services, , other
   3. How involved do you feel/would you like to feel in making decisions about your care with these people/services?
8. Do you have any suggestions on how EI services can be improved?
   1. What would an ideal EI service look like to you?
      1. Access to service
      2. Discharge from service
      3. Duration
      4. Content of service – what more would you have liked? What could be done differently? (mental and physical health)
9. Are there any positive or negative examples of EI services you would like to share? What contributed to a good or negative experience of EI services?
10. Do you have anything you wish to add?

**End of interview**

Thank you for participating.

Arrange reimbursement.

Would you be happy for us to keep your details and contact you in the next stage of the study?

Ask participant if they would be interested in receiving a summary of findings (and how we should communicate this).

**Close**

**TOPIC GUIDE - CARER**

**Introductions**

Answer any questions from participant

Complete consent process – including consent to record.

**Background information**

- Relationship to SU
- gender, age, ethnic background, family circumstances, housing, employment, other physical and mental health diagnoses

**Interview topics to be covered**

1. How long has the person you provide support for been in the EI service?
   1. Explore their experiences which led to original referral
   2. Explore current challenges (for SU and carer)
   3. Explore carer’s hopes for the future
2. Can you outline the support/treatment your family member/loved one has received from the EI service?
   1. Who was most helpful and why?
   2. What help did they provide?
3. Did you or anyone else in your family receive support from EI service?
   1. If so, what?
   2. If not, what might have been useful?
   3. What were your hopes for support from the EI service?
4. How do you feel EI service has helped the person you care for ?
   1. Mental health
   2. Physical health
5. Do they feel equipped to move forward with their lives?
6. Has the Ei service supported you to support the person you care for?
   1. Do you feel equipped to move forward?
7. What preparation and support did the person you care for receive from the EI service for discharge?
   1. Mental health and physical health
   2. What support did you (and your family) receive?
   3. How was the person you care for involved in the decision about discharge?
      1. How were you involved in the decision about discharge? Would you have liked to be more involved in the decision-making process? How did you feel about discharge?
   4. How was the person you care for involved in planning their discharge and the services they might have following discharge from the EI service?
      1. How were you involved?
      2. Were any ongoing support needs of family considered?
8. What services have been put in place to support the person you care for following discharge? What services have been put in place to support you?
   1. Explore health support CMHT GP – both physical and mental health support
   2. Explore role of social prescribing, housing support, social services, other
   3. How involved do you feel/would you like to feel in making decisions about your loved one’s care with these people/services?
9. If the person you care for was offered further support from the EI service – what would that look like? If you, as a carer, were offered further support, what would this look like?
10. Do you have any suggestions on how EI services can be improved?
    1. What would an ideal EI service look like to you?
       1. Duration
       2. Care provided – what more would you have liked? What could have been done differently?
11. Are there any positive or negative examples of EI services you would like to share?
12. Do you have anything you wish to add?

**End of interview**

Thank you for participating.

Arrange reimbursement.

Ask participant if they would be interested in receiving a summary of findings (and how we should communicate this).

**Close**

**TOPIC GUIDE – SERVICE USER (SU) FOLLOW UP**

**Introductions**

Thank you for agreeing to be interviewed for a second time

Answer any questions from participant

Complete consent process – including consent to record.

Take GP details (with explanation why)

**Confirm background information**

- Gender, age, ethnic background, family circumstances, housing

**Interview topics to be covered (informed by the first interview)**

1. How have things been for you over the last 6 months?
2. What services have you engaged with during the time since your discharge from the EI service?

- Have you had any contact with your GP/practice? Is this for your mental health or physical health? Or both?
- Have you had any contact with mental health services? Is this for mental health or physical health or both? Have you been admitted to mental health services? Or seen in ED/ Home Based Treatment Service?
- How easy were these services to access?
- Have you had any contact with social services? Third sector services/social prescribing?
- Have you had any contact with any other services? (eg Police)

1. What has been the most helpful support you have received? Can you explain how it is/was helpful?
2. Are there any services you would like to receive support from but haven’t accessed? Why? How could this be made more accessible?
3. What services have your family received support from? How easy was this support to access?
4. What is the most helpful support your family has received (if any)? How is/was it helpful?
5. Are there any types of support your family would like but haven’t been able to access? Why? How could this be more accessible?
6. How do you feel now about your situation? Do you feel that you have ‘recovered’? what does ‘recovery’ mean to you?
7. Looking back, do you have any further thoughts on how EI services could be improved?
   - - Are there any types of care you would have liked to receive but didn’t?
     - How do you now feel about the length of care you received?
     - How do you feel about the discharge process?
8. Do you have anything you wish to add?

**End of interview**

Thank you for participating.

Arrange reimbursement.

Ask participant if they would be interested in receiving a summary of findings (and how we should communicate this).

Confirm if participant would be willing to suggest other people who support them to be interviewed and explain the process.

**Close**

**TOPIC GUIDE –HEALTHCARE PRACTITIONERS**

**Introductions**

Answer any questions from participant

Complete consent process – including consent to record.

**Background information**

- Invite participant to briefly describe their role
  - How long have they been in this role?
  - For GPs:
    - partner (principal), salaried GP, locum
    - any special interests
    - size of practice
    - rural/sub-urban/urban/inner city
    - gender, age (range), ethnicity

**Interview topics to be covered**

1. Please describe your role in the care of people with severe mental illness.
2. Please describe your role in relation to the EI service.
3. Can you explain how the EIP service works?
   1. How are people referred in?
   2. What is duration of care?
   3. What sort of care do people within the EIP service receive?
   4. How are discharges managed?
4. Can you describe a patient who was recently discharged from EI service (obviously I don’t need the name of the patient).
   1. Outline their problems (mental health diagnosis; any physical health problems)
   2. Outline support/management given by the EI service to this person
   3. How did the EI service support the family?
   4. Can you describe how the decision to discharge the person was made?
      1. Is this how decisions were made usually? (if so, explore; if not, why not)
      2. How do you involve SUs and their carers in the decision to discharge from your EI service?
5. How long do people usually stay in the EI service?
   1. What dictates that time?
6. Are there circumstances when you think that a service user should stay in EI service but you are not able to offer this?
   1. If yes, explain why and please give examples – what would further support/management look like?
   2. If not, please explain
7. How are plans made to support a SU after discharge from EI service?
   1. How are SUs and their families involved in making these plans?
   2. What sort of liaison goes on with GP/primary care?
   3. How confident do you feel that these plans work out?
   4. Which services will be built into management plan following discharge? (GP, CMHT, third sector, social prescribing other)
8. How confident do you feel when a person is discharged that the plans are followed?
   1. When they are not – why not?
9. Do you feel that a SU needs to be ‘recovered’ in order to be discharged from the EI service?
   1. What do you feel the term ‘recovery’ means?
10. Do you have any suggestions on how EI services can be improved?
    1. What would an ideal Ei Service look like?
11. Are there any positive or negative examples of EI services you would like to share?
12. What impact did the COVID-19 restrictions have on the service you offered?
    1. Explore situation pre-pandemic, 2020, 2021, 2022….
13. Do you have anything you wish to add?

**End of interview**

Thank you for participating.

Ask participant if they would be interested in receiving a summary of findings (and how we should communicate this).

**Close**

**TOPIC GUIDE - COMMISSIONERS**

**Introductions**

Answer any questions from participant

Complete consent process – including consent to record.

**Background information**

- Invite participant to briefly describe their role
  - How long have they been in this role?
- Gender, age (range), ethnicity

**Interview topics to be covered**

1. Please describe your role in relation to commissioning MH services, and in particular the EI service.
   1. Explore where employed – PCN or ICB – and have there been any changes in role due to recent organisations?
   2. How long have you been in this role? What have you done in recent past?
2. Can you describe your local EI service?
   1. Referral criteria
   2. Treatment and support offered
3. Are you aware how long people usually stay in the EI service?
   1. What dictates that time?
   2. Do you think it should be longer/shorter? Why?
4. Are there circumstances that you are aware of when you think that a SU should stay in EI service but you are not able to offer this?
   1. If yes, can you give any examples – what would further support/management look like?
   2. If not, please explain
5. Do you know are plans made to support a SU after discharge from EI service?
   1. How are SUs and their families involved in making these plans?
   2. Which services will be built into management plan following discharge? (GP, CMHT, third sector, social prescribing, other)
   3. Are plans made to monitor physical health after discharge?
6. How confident do you feel when a person is discharged that the plans are followed?
   1. When they are not – why not?
7. Do you feel that a SU needs to be ‘recovered’ in order to be discharged from the EI service?
   1. What do you feel the term ‘recovery’ means?

1. How are the lay public involved in MH commissioning, and in particular EI services?
   1. Have you received any lay feedback on current services?
2. Do you have any suggestions on how EI services can be improved?
3. Are there any positive or negative examples of EI services you would like to share?
   1. Either the service you commission or services around the country you are aware of
4. What impact did the COVID-19 restrictions have on the Ei service you commissioned?
   1. Explore situation pre-pandemic, 2020, 2021, 2022….
5. Do you have anything you wish to add

**End of interview**

Thank you for participating.

Ask participant if they would be interested in receiving a summary of findings (and how we should communicate this).

**Close**
